# Development of a Low-Cost Arduino-Based Sonde for Coastal Applications

**DOI:** 10.3390/s16040528

**Published:** 2016-04-13

**Authors:** Grant Lockridge, Brian Dzwonkowski, Reid Nelson, Sean Powers

**Affiliations:** 1 Dauphin Island Sea Lab., 101 Bienville Blvd., Dauphin Island, AL 36528, USA; briandz@disl.org (B.D.); tnelson@disl.org (R.N.); spowers@disl.org (S.P.); 2 Department of Marine Sciences, University of South Alabama, Life Sciences Building Room 25, Mobile, AL 36688, USA

**Keywords:** Arduino, drifter, data logger, CTD, physical sampling, Mobile Bay, marine, low-cost

## Abstract

This project addresses the need for an expansion in the monitoring of marine environments by providing a detailed description of a low cost, robust, user friendly sonde, built on Arduino Mega 2560 (Mega) and Arduino Uno (Uno) platforms. The sonde can be made without specialized tools or training and can be easily modified to meet individual application requirements. The platform allows for internal logging of multiple parameters of which conductivity, temperature, and GPS position are demonstrated. Two design configurations for different coastal hydrographic applications are highlighted to show the robust and versatile nature of this sensor platform. The initial sonde design was intended for use on a Lagrangian style surface drifter that recorded measurements of temperature; salinity; and position for a deployment duration of less than 24 h. Functional testing of the sensor consisted of a 55 h comparison with a regularly maintained water quality sensor (*i.e.*, YSI 6600 sonde) in Mobile Bay, AL. The temperature and salinity data were highly correlated and had acceptable RMS errors of 0.154 °C and 1.35 psu for the environmental conditions. A second application using the sonde platform was designed for longer duration (~3–4 weeks); subsurface (1.5–4.0 m depths) deployment, moored to permanent structures. Design alterations reflected an emphasis on minimizing power consumption, which included the elimination of the GPS capabilities, increased battery capacity, and power-saving software modifications. The sonde designs presented serve as templates that will expand the hydrographic measurement capabilities of ocean scientists, students, and teachers.

## 1. Introduction

Observations of water conditions in the coastal marine environment are critical for a range of management applications as well as improving the understanding many estuarine and near-shore processes. Basic measurements of temperature and salinity are important to resource managers due to the sensitivity of certain commercially important species as well as determining weather conditions are favorable for the presence and/or rapid growth of pathogenic organisms. For example, low salinity and high temperatures have been shown to impact the metabolism and overall health of oysters (*Crassostrea virginica)*, both synergistically and independently [1]. In addition, extreme temperatures around oyster reefs can increase the likelihood of *Vibrio* outbreaks, a pathogen that causes human illness, and in some cases, death [2]. From a scientific perspective, temperature and salinity are key parameters that impact coastal processes from metabolic rates to circulation patterns. Coastal systems often have large temporal and spatial gradients in abiotic properties, which can require site specific observations.

The dynamic and corrosive nature of the marine environment can present a substantial challenge to resource managers and scientists attempting any type of environmental monitoring [3]. The typical solution is to use expensive, highly specialized instrumentation and proprietary software developed by companies such as SeaBird Electronics Inc. or Xylem Inc. While these devices are generally reliable, their cost often prevents monitoring at optimal spatial resolutions, with each instrument requiring a substantial financial investment. Additionally, monitoring efforts are limited only to a select few individuals with sufficient funding, often inhibiting educational groups and general citizens, as well as scientists with constrained resources, from these efforts/projects. The development of a reliable, low cost, customizable alternative to current hydrographic monitoring instrumentation would make data collection feasible for anyone interested in the coastal environment, and also allow established researchers to increase the resolution of their existing sampling efforts [4].

Historically, only trained engineers possessed the expertise and tools necessary to manufacture instruments capable of sampling hydrographic parameters with acceptable accuracy, precision, and resolution reliably. However, in the past 10 years, integrated circuit microcontrollers (ICM) have provided a platform that can be developed into a customizable, robust, low cost data logger [5]. Even more recently, the Arduino microcontroller platform has surfaced as a user-friendly version of ICM, specifically designed for people with little or no background in electronics or programming. The easy to use software is free to download online, and supported by an expanding open-source online community [6]. The hardware is inexpensive, the base model, Arduino Uno, can be purchased for around $25 US, and can be combined with any number of sensors and/or instruments that are available from a variety of retailers (e.g., Adafruit, Atlas Scientific). With minimal development effort or experience, it is feasible that tools capable of high resolution environmental monitoring could be created without a large financial investment. The Arduino platform was chosen over similar alternatives (*i.e.*, Raspberry Pi, Gumstix) because of its lower price and the extensive online support network. Additionally, the Arduino was found to be capable of withstanding a large amount of physical abuse, making it the ideal option for deployment in harsh marine environments.

The use of open source microcontrollers for data collection is a trend that has been increasing in popularity in the past few years. The utility of the Arduino based microcontroller is clearly evident and viable, with examples of their incorporation ranging from automated sterilization of laboratory equipment [7] to *in situ* measurements of phytoplankton florescence [8]. While generally reported as successful, insufficient resolution is the most commonly noted limitation of these electronics [8,9,10]. However, the resolution limits, while not ideal for all applications, are sufficient for many efforts, namely, environmental monitoring or educational endeavors, where the low cost and the ability to manufacture and customize multiple units take precedence over higher precision data collection. Other similar efforts have utilized Raspberry Pi and Gumstix platforms successfully [11], and while they have more powerful processors, and can interface with a wider range of sensors and instruments, they require more expertise and are less cost effective than the design described here.

The majority of the low cost designs are confined to “above water” environments [10,12,13,14,15,16], likely because development of a completely waterproof electronics housing with external probes can be challenging and expensive. The authors of [17] describe an Arduino based data logger for ecological applications, and report successful deployments and data collection in rocky intertidal environments, but the design was not meant to be completely submerged. However, [8] successfully developed an underwater housing, but the parameter they were measuring, florescence, does not require a probe with direct access to the outside, and can be measured through a clear panel. Similarly, Busquets *et al.* [18] describe the development and early testing of an automated underwater vehicle, controlled with an Arduino Mega, but all sensing was confined to inside the structure of the vehicle’s body. Parameters such as conductivity present the unique challenge of needing direct access to the outside environment, while also requiring a complete, waterproof, seal between the probe’s cable, or body, and the electronics’ housing wall. Waterproof electrical bulkheads are expensive, and as a result, projects that have successfully incorporated conductivity into off-the-shelf microcontroller applications [11], place less emphasis on minimizing cost. Our design, in addition to the sonde itself, includes a low-cost, waterproof housing, built from off-the-shelf hardware, capable of monitoring physical parameters (*i.e.*, conductivity and temperature) in marine environments, while deployed underwater to depths up to 4 m. Nevertheless, the prevalence of Arduino based microcontrollers in the literature indicates that the development of low-cost sondes and data loggers is now possible and appealing to a wide range of professionals. As such, the capabilities and limitations of this new technology need to be explored as thoroughly as possible in order to be more widely accepted and utilized.

The objective of this project was to determine if an Arduino based sonde, comprised of low-cost, off the shelf components, is capable of supporting the physical monitoring needs of a variety of coastal scientific applications. This paper describes the development process and functional testing of the initial sonde design, and also details modifications that were made to the sonde, based on the needs of two independent coastal applications, and the results from the initial deployments. The focus on coastal applications derives from two different projects examining processes that require a low-cost means of measuring temperature and salinity in the marine environment. One of the projects that used this sonde is examining aspects of estuarine-shelf exchange at the mouth of a tidal inlet that is strongly influenced by river discharge. This work is mapping the transport pathways and entrainment rates using Langragrian drifters in order to better understand the dispersion of estuarine waters on the shelf. The other application driving this sensor development involved tracking fish movement. This work focused on collecting environmental data (salinity and temperature) in conjunction with an acoustic fish tracking array in order to relate fish movements to changing abiotic parameters. Thus, both variants of the sonde design stem from a scientific need on active coastal research projects, illustrating the direct application of this sonde.

## 2. Materials and Methods

### 2.1. Sonde Design and Functional Testing

In order to illustrate the customizability of the sonde design, two applications were tested. In the first, the sonde was programmed and outfitted with hardware that allowed it to record position, temperature and conductivity every 60 s, and was deployed in a free floating lagrangian drifter housing. The second design eliminated the GPS hardware, and recorded temperature and conductivity every 30 min. This second sonde design was deployed underwater to a maximum depth of 4 m, and was left for 24 days. Overlapping design characteristics are described first, followed by unique features of the drifter and moored applications.

**Figure 1:**
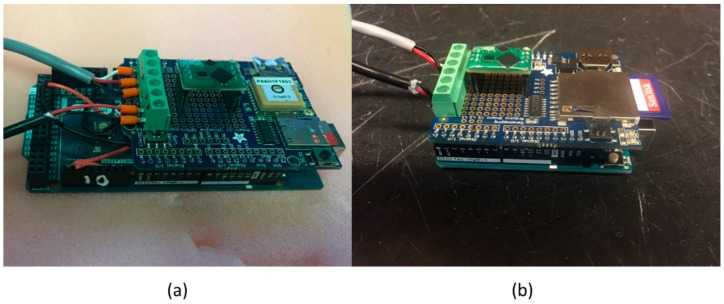
Assembled Arduino based sonde for (**a**) the drifter application and (**b**) the moored application.

The design of the sonde was based around the Arduino platform (either Mega 2560 or Uno) with a data logging attachment (either the Adafruit Ultimate GPS Logger Shield (AUGPSS) or the Adafruit Data Logging Shield). The assemblies of both iterations can be seen in Figure 1a,b. Serial communications were established between the microcontroller and datalogger, and an Atlas EZO EC microchip (EC) was installed in the available prototyping space. Temperature and conductivity probes were subsequently connected with screw terminals and wired according to manufacturers (Atlas Scientific) recommendations. The temperature probe used serial communications to report while the EC and conductivity probe used I2C with an address of 100. The program included a timer function using the millis() library, allowing sampling intervals to be customized by the user [6]. At specified sampling intervals, the raw temperature was acquired from a voltage drop, which was used to calculate the temperature in Celsius. This temperature was then sent to the EC microchip, to be used for temperature compensation of the conductivity reading. The EC microchip received a voltage from the conductivity probe, which was converted into conductivity, salinity, specific gravity, and total dissolved solids. Temperature and conductivity readings were recorded to the SD card as a comma separated values (CSV) file. Collected data were then uploaded from the SD card to a computer where they were analyzed. Prior to deployments, temperature was calibrated to a YSI 2030 pro model handheld sonde, and conductivity was two point calibrated using 12,880 µS and 80,000 µS standard solutions. Modifications to this general design were made to meet the specific deployment requirements for the drifter and moored applications

**Figure 2:**
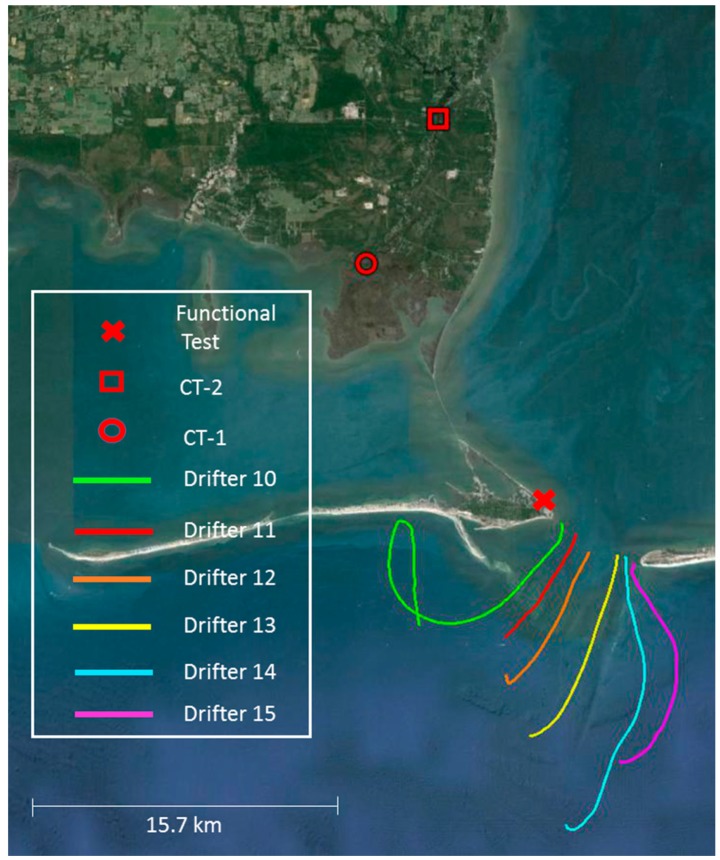
Plots of the recorded GPS tracks from the six drifters deployed to test the drifter application are represented by colored lines. The location of the functional testing is marked with a red X. The red square and circle identify the locations of the moored application deployments.

Functional testing consisted of submerging the sonde in a drifter frame without a drogue to a depth of 5 m. A measurement was taken every 60 s over the deployment, which occurred from 1546 GMT 24 August 2015 to 1146 GMT 27 August 2015 at the Dauphin Island Sea Lab (DISL) Weather Station located at 30°15.075N, 88°04.670W, marked in Figure 2 with a red ‘’X”. The deployment was in close proximity (<10 m) to a logging YSI 6600 sonde, allowing for data validation by comparing readings from the two instruments. Additionally, these data provided a battery life estimate for future deployments.

### 2.2. Drifter Application

#### 2.2.1. Drifter Hardware

For the drifter application, an Arduino Mega 2560 (Mega) was used with the AUGPSS. Pin 8 on the AUGPSS was jumped to pin 52, because pin 10, the default on the AUGPSS, does not support serial communication on the Mega. The AUGPSS comes supplied with a PA6H GPS module, microSD card read/write slot Real Time Clock (RTC) with coin cell battery backup, and a prototyping area. In the prototyping area, the EC was installed and the PRB connections were attached to a three terminal header block. This terminal block serves as the connection for the Atlas Scientific K1.0 Conductivity Probe. The EC VCC pin was soldered to the 5 volt pin on AUGPSS and the GND pin on the EC was soldered to the ground pin on the AUGPSS. The TX and RX pins on the EC were connected to pins 20 and 21 on the Mega, respectively, which support SDA and SCL communications used to communicate with the EC. Finally, a second three slot terminal block was added to the AUGPSS prototyping area. One terminal was connected to ground on the Mega, one terminal was connected to pin 50 on the Mega, and one terminal was connected to pin A8 on the Mega. This terminal serves as the connection for the Atlas Scientific ENV-TMP temperature probe. The drifter application was powered with six C-cell batteries wired in series producing 9 volts. The decision to use alkaline C-cell batteries was based on the physical design of the housing and adherence to the low cost component of the design. Specifically, six C-cell batteries oriented in the electronics housing of the drifter, fit snugly inside of the standard PVC pipe, and resulted in the correct amount of ballast to achieve the desired buoyancy and orientation in the water, with minimal adjustments. Additionally, the likelihood of losing a drifter was high, and while alternative power supply options would likely have resulted in longer deployments capabilities, if lost, their replacement would substantially increase the project’s cost.

#### 2.2.2. Drifter Software

**Figure 3:**
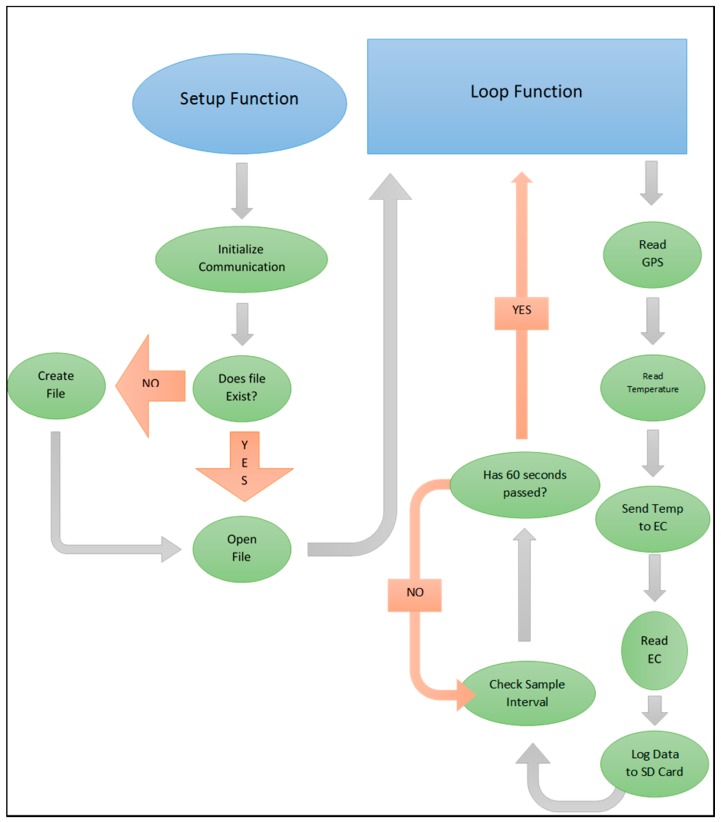
Programming logic followed by the sonde used for the drifter application.

The code for the drifter application was written with the Arduino integrated development environment (IDE) software, which was based on the C++ language. The IDE was downloaded for free from the Arduino website. Source code used for the drifter and moored applications of the sonde can be found in Supplementary Materials. The code for the drifter application was compiled from the open source support available online, and followed the logic depicted in Figure 3. Sampling interval for the drifter application was set to 60 s, but could be user specified to a 1 ms interval. GPS data were received, filtered for the most recent NMEA sentence, then parsed into integers and saved to the microSD card as Latitude, Longitude, Speed, Number of Satellites Locked, Date, and Time. Temperature and GPS readings were transferred through serial channels at 9600 baud, while conductivity used I2C communications with the EZO-EC which had a default address of 100. The temperature and conductivity probes were supplied with 5 volts, which was reduced by the interaction of the probe with the environment, the resulting voltage change represented the raw parameter measurement. The standard resolution of the Arduino Mega 8 bit processor was 1023 intervals, which when applied to the range of the temperature sensor (−20 to 133 °C) resulted in approximately 0.25 °C. Resolution could be increased with the addition of an amplifier (*i.e.*, INA125P), but was not necessary for this project. However, the EZO-EC performed the function of an amplifier, and provided a conductivity resolution of 0.01 µS over a range of 0.07–99.99 µS with the setup described. In addition to amplification, the EZO-EC temperature compensated and calculated salinity, total dissolved solids, conductivity and specific gravity. In between the sampling interval, the EZO-EC was put into a quiescent state to minimize power consumption. The data were saved as a CSV file on the microSD card. If power was removed or lost, the new data were appended to the existing file, if present, when power was re-supplied. A new file was created if none existed on the microSD card.

#### 2.2.3. Drifter Housing

**Figure 4:**
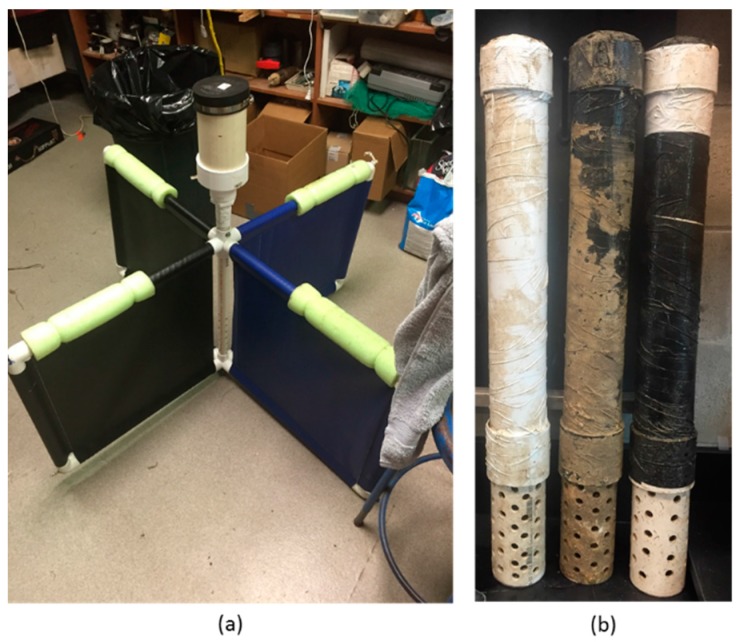
Picture of the deployment housing for (**a**) the drifter application and (**b**) the moored application.

The drifter application was developed to be deployed in a lagrangian drifter following the industry accepted 40:1 Drag Area Ratio (DAR) described by [19] Figure 4a. The drifter frames were constructed from 2.54 cm Polyvinal chloride (PVC) tubes and consisted of a collapsible drogue, underwater sensor housing, and waterproof compartment to house the sonde electronics.

The drogue contained four panels, each measuring 68.5 cm wide × 58.5 cm deep, and were oriented in a cross. Each panel was covered with heavy duty nylon fabric that slipped onto three of the four PVC frame sides. The four panels share the central vertical PVC support, utilizing 5-way and 6-way 2.54 cm PVC fittings. The 5-way fitting was fitted with a ¼-20 stainless steel bolt and nut, to which lead weights were added for ballast. The central vertical PVC support was drilled with numerous, haphazardly placed 5 mm holes, and housed the temperature and conductivity probes. Two of the drogue panels directly across from each other were glued in place, while the remaining two were secured with 7 mm heavy duty bungee cord, stretched along the upper and lower long axes and passed through 1 cm holes in each outer elbow, where it was secured with an overhand surgeons knot. This design was intended to allow for more drifters to be loaded onto a small boat at once, facilitating easier deployment logistics.

The waterproof compartment was attached to the drogue with a ¼-20 stainless steel bolt to allow the compartment to be easily disconnected for transportation, data retrieval, and calibration. Beginning at the bottom, a stainless steel washer (ID = 1.7 cm; OD = 3.36 cm) was placed in the slip end of a 2.54 cm threaded female PVC adapter. The washer dimensions are critical to ensure that it will fit inside the female adapter while allowing the probes to pass through the middle. An 8 cm long piece of 2.54 cm was then glued on top of the washer, permanently securing both in place. A tapered round rubber plug (3.175 cm top; 2.54 cm bottom; 2.54 cm height), with two 4.5 mm holes bored through its center, was placed large end first into the threaded end of the female threaded adapter. The probe cables were then passed through the washer and plug leaving approximately 40 cm from the top of the probes to the bottom of the rubber plug. A threaded male 2.54 cm adapter was then screwed into the female adapter making sure that the rubber plug fit was secure and the probe cables remained unkinked. This design creates a watertight seal on the probe cables using the compression of the rubber plug and was tested to 5 m without the detection of leaks. The slip end of the threaded male adapter was then glued to a 5.08 cm × 2.54 cm reducing PVC bushing, which was glued into a 10.16 cm × 5.08 cm reducing PVC coupling. A 22 cm long piece of 10.16 cm PVC pipe was then glued into place. This creates the main body of the waterproof compartment. Inside the compartment, a pack of 6 C-cell batteries, wired in series, was placed at the lowest point. The probes were then attached to the labeled screw terminals on the data logger, which was then wrapped in non-conductive foam to minimize impact damage to the sensitive electronics. The top of the compartment was sealed with a 10.16 com rubber cap with a stainless steel worm-drive hose clamp. The rubber cap provides water resistance to 5 m, while still allowing the exchange of GPS position data while exposed to the surface.

#### 2.2.4. Drifter Deployment

Six drifters, outfitted with this sonde configuration, were deployed systematically across the mouth of Mobile Bay, Alabama at slack high tide on 4 September 2015. The drifters were set to sample every 60 s and were allowed to disperse for approximately 6 h before recovery. GPS data were imported to Google Earth for visual representation. The data were used to evaluate drifter performance and assess plume characteristics.

### 2.3. Moored Application

#### 2.3.1. Moored Hardware

The moored application of the sonde uses an Arduino Uno in order to minimize cost, size, and battery consumption. The sonde was outfitted with an Adafruit Data Logging Shield which contains a SD card reader slot, a RTC with coin cell backup battery, and a prototyping area. The data logging shield replaced the GPS shield that was used for the drifter application, as the moored application was designed to be attached to a permanent structure at a known location, making position acquisition data unnecessary. This also reduced the power consumption of the sonde, helping to extend the deployment time. The EC was installed similarly to the drifter application hardware, with the PRB connections attached to a three terminal header block that serves as a connection for the K1.0 Conductivity Probe. All power pins were connected in the same way as the drifter application, however the TX and RX pins were connected to pins A4 (I2C SDA) and A5 (I2C SCL), respectively on the Uno. A three slot terminal block was added for the ENV-TMP temperature probe with the temperature terminal block wires connecting GND, A0, and D4 on the UNO to the black wire (negative), white wire (signal), and red wire (positive) on the temperature probe. The moored application was powered by two battery packs wired in parallel consisting of six D-cell batteries producing approximately 9 volts. The D-cell battery packs provided enough ballast to easily submerge the housing, making deployment easier, while also making longer deployments possible by increasing the ampere hours from the drifter design. Additionally, if the housing were to flood, the cost of the replacement of the power supply would be minimal.

#### 2.3.2. Moored Software

**Figure 5:**
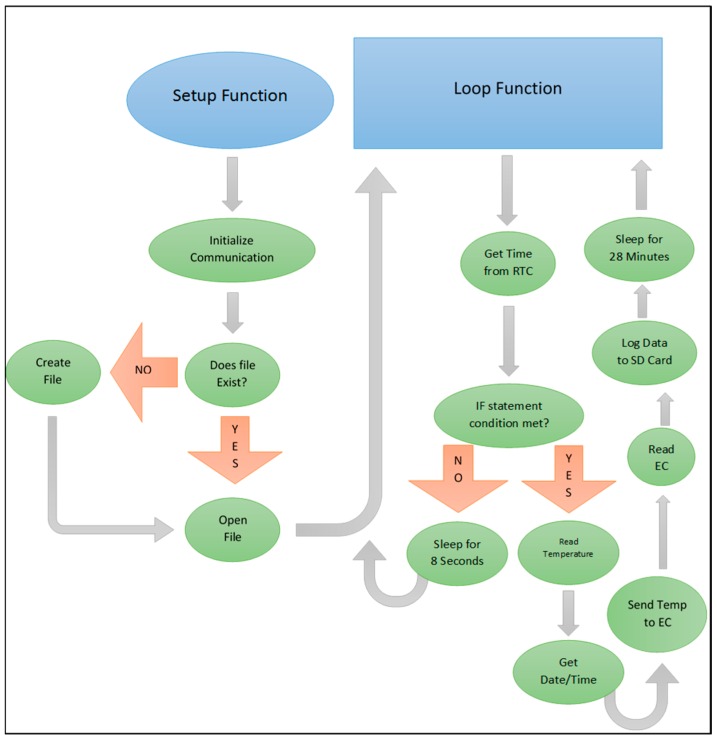
Programming logic followed by the sonde used for the moored application.

The code for the moored application was compiled using the Arduino IDE environment and open source coding available online and follows logic depicted in Figure 5. The function of the moored application is for a relatively long term field deployment so the sampling interval was set to 30 min using an IF statement coupled with the real time clock (RTC). This allowed for a sample to be logged every half hour in order to mimic other long-term hydrographic monitoring occurring throughout Mobile Bay using the RTC for internal timing. In order to minimize power consumption the LowPower.powerDown function from the LowPower Library was used [20]. This function uses the built-in watchdog timer (WDT) on the Arduino and it can be set to a maximum of 8 s. In order to achieve a longer sleep time, this command can be put into a WHILE statement, allowing it to be repeated as many times as necessary. Given that a 30 min delay between samples was achieved with the RTC IF statement, the 8 s power down function was repeated 210 times, resulting in a sleep interval of 28 min after each sample. After this interval the processor would awaken and check to see if the conditions of the if statement were met (minute = 0 or minute = 30), if so it would log a sample and then sleep for another 28 min, if not, it would go to sleep for 8 s and then awaken and check conditions of the if statement. Given that sample logging is not instantaneous the 28 min interval was used instead of an even 30 min delay in order to ensure that a sample was logged every half hour. Headers for the data columns were shortened and stored in the flash memory on the UNO given that the other memory banks on the UNO are quite small. All data were saved as a .CSV file on the SD card.

#### 2.3.3. Moored Housing

The moored application was developed to be similar to a YSI 6600 and is intended for long term (3–5 weeks) underwater field deployment. It consists of a watertight housing for the sonde electronics and a probe cage for protection during deployment. The main waterproof housing was constructed with a 56 cm long piece of 7.62 cm PVC pipe. This piece of pipe was capped at the bottom with a 7.5 cm Oatey Gripper PVC plug that had holes drilled through the plastic plug and a rubber punch was used to create two holes in the seal for the probe wires to pass through. When compressed into the end of the pipe this plug created a watertight seal in the pipe and around the probe wires without kinking them. Given that this plug is compressed with a wingnut, enough wire was left on the outside of the plug so that the probes did not interfere with the wingnut. In order to prevent biofouling both probes were wrapped in copper tape and the conductivity probe opening was covered with a 0.305 mm mesh copper screen. A cage was constructed to cover the probes and consisted of a 21 cm piece of PVC pipe capped on the bottom, with multiple 1.27 cm holes drilled haphazardly in the pipe. This cage was glued into a 7.62 cm PVC coupler and slid onto the housing (unglued) creating a tight attachment for the cage while ensuring that the plug remained in the pipe.

Inside the housing the 12 D-cell battery pack rested on the bottom plug and the sonde electronics hardware was placed above the pack at the top of the housing. All components inside the housing were padded with paper towels to minimize movement and impact between pieces. The top of the housing was capped with another Oatey plug (unaltered) and both plugs were greased with silicon grease to ensure that a watertight seal was achieved. The housing was then capped with a 7.62 cm PVC end cap (unglued) to protect the plug and minimize incidental damage. The completed mooring application housing can be seen in Figure 4b.

#### 2.3.4. Moored Deployment

Two sondes were used for the moored application, CT-1 and CT-2. Each was attached to permanent pilings using two large zip ties and one ratchet strap. CT-1 was deployed 1.5 m depth, while CT-2 was deployed at 4 m. Deployment locations are depicted in Figure 2 as a red “O” (CT-1) and red “□” (CT-2). Each housing was wrapped in saran wrap and duct tape before deployment so that any biofouling on the housing could be easily removed. These data are currently being used to determine abiotic characteristics of a tidally influenced river.

### 2.4. Construction Costs

The cost of each component for the drifter and moored applications as well as the total is presented in Table 1. The total cost of the drifter application design was $306.00 US, while the total cost of the moored application design was $255.80 US.

## 3. Results

### 3.1. Functional Testing

**Figure 6:**
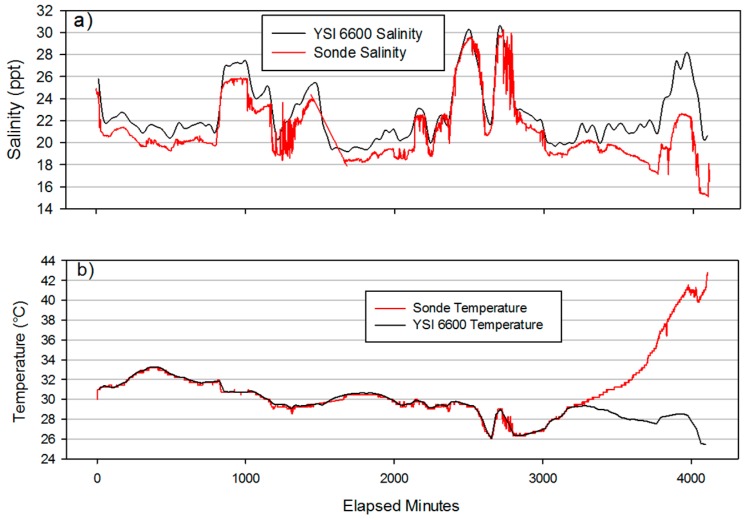
Comparison of salinity (**a**) and temperature (**b**) between the drifter configuration of the sonde and the Dauphin Island Sea Lab (DISL) Weather Station YSI 6600 from 1546 GMT August 24th 2015 to 1146 GMT August 27th 2015.

Functional testing was conducted from 1546 GMT 24 August 2015 to 1146 GMT 27 August 2015 at the Dauphin Island Sea Lab (DISL) Weather Station located at 30°15.075N, 88°04.670 W. Results are presented in Figure 6a,b. All comparisons were limited the first 55 h of the deployment to eliminate erroneous readings that resulted from depleted battery power. Salinity measurement comparisons between the drifter configuration and a YSI 6600 sonde found RMS error to be 1.35 ppt and temperature measurement RMS error to be 0.154 °C. While the salinity difference was higher than anticipated, this error was only approximately ten percent of the observed salinity range and appeared to be a slight offset/bias that is likely of a physical nature (See Section 4.1). Regressions were also used to compare salinity and temperature readings between the two devices and found to be highly correlated for both parameters (r^2^ = 0.96 for salinity and r^2^ = 0.99 for temperature). Battery power lasted for 54 h and 43 min before voltage dropouts resulted in deviations. This can be viewed in Figure 6 as a separation of salinity and temperature measurements that occurs around 22:00 GMT on 26 August 2015 at reading 3100. 

### 3.2. Drifter Deployment

**Figure 7:**
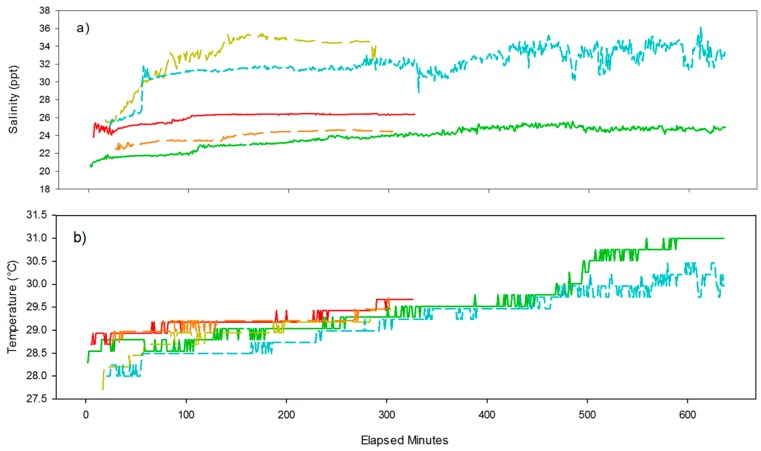
Salinity (**a**) and temperature (**b**) readings from drifters 10–14 during deployment on September 4th 2015 across the mouth of Mobile Bay, Alabama. The color scheme is the same as Figure 2.

A total of six drifters were deployed on 4 September 2015 across the mouth of Mobile Bay, Alabama. The tracks that were recorded by the drifters are shown in Figure 2. The drifters were released between 11:16 and 11:41 GMT. Recovery times ranged from 4 h 22 min (drifter 12) to 24 h 26 min (drifter 10). The distance each drifter traveled can be referenced in Table 2, as well as temperature and salinity measurements from a handheld YSI 2030 and the drifters upon recovery. Salinity and temperature measurements from the drifter deployment can be found in Figure 7a,b. A temperature calibration error was discovered in drifter 15 post deployment and as a result, no data from drifter 15 are shown in Figure 7.

### 3.3. CT Deployment

**Figure 8:**
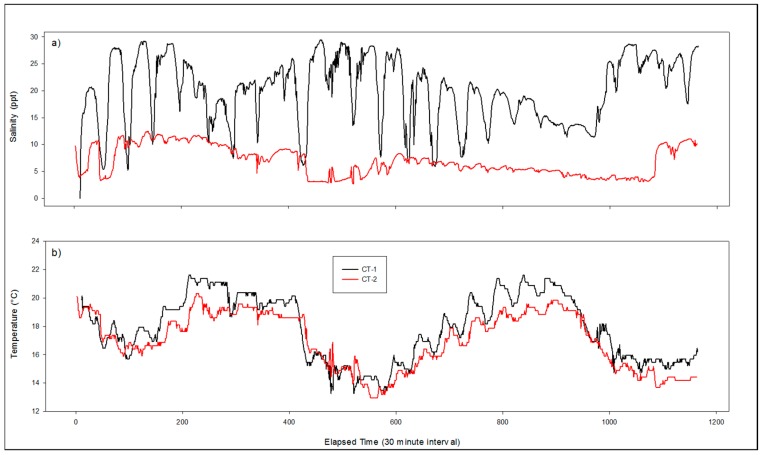
Salinity (**a**) and temperature (**b**) measurements taken with the moored application design from sites CT-1 and CT-2 during 13 November to 7 December 2015 deployment.

Two conductivity and temperature data loggers (CT) using the moored application design were deployed for a total of 24 days from 13 November 2015 to 7 December 2015. CT-1 was deployed in West Fowl River (30°21.742 N, 88°10.434 W), while CT-2 was deployed at Bellingrath Gardens (30°25.716 N, 88°8.138 W). Both locations can be seen in Figure 2 above. Salinity and temperature measurements were taken every 30 min and the results can be viewed in Figure 8. Salinity was consistently higher at the CT-1 site while temperatures at both CT-1 and CT-2 tracked similarly over the entire deployment.

## 4. Discussion

### 4.1. Functional Testing

In general, the functional testing indicated that the performance of the sonde was adequate for the expected environmental conditions and the level of accuracy needed. Conductivity measurements were consistently lower than those recorded by the YSI 6600 during functional testing. There are physical reasons that could explain the difference between the two instrument records. For example the physical separation (approximately 0.5 m) between the instrument sensors may have caused the experimental sonde to have the observed lower salinity bias. Mobile Bay is a highly stratified environment that commonly experiences vertical gradients in salinity associated with freshwater discharge that are modulated by wind driven mixing events [21,22]. However, it is unclear if this is in fact the case as vertical stratification is dominated primarily by thermal density changes in the summer [22], and so a temperature offset would be expected to accompany the observed salinity offset.

Alternatively, instrument biofouling is a significant issue during the summer along the Northern Gulf Coast, and commonly results in sensor drift after 3–4 weeks of deployment [23]. Carbonate and organic structures that comprise the majority of biofoulant, are capable of interfering with a sensors conductivity measurements, while having no significant effect on thermal conductance, and do not impact temperature measurements [24]. The YSI 6600 used for comparison during functional test one was deployed on 9 August 2015 and had accumulated 15 days of growth. This is the most likely explanation for the discrepancy between the two instrument’s salinity measurements. However, for the two applications using this design, in an environment known to fluctuate 20 psu in a single tidal cycle, an RMS error of 1.35 psu was determined to be acceptable, so no further testing or design refinements were attempted. It is likely possible to achieve higher resolution salinity measurements with Arduino based sondes than those reported here, however, applications requiring better precision should either be prepared to explicitly test the sonde setup to accuracy thresholds that meet project requirements, or explore the use of alternative hardware combinations than the Arduino and conductivity sensors used here.

Temperature comparisons between a YSI 6600 and the drifter application design indicate excellent tracking between the measurements. No significant difference between the mean differences was present (*p* < 0.01). The divergence between sondes occurred around 2200 GMT on 27 August 2015 (Figure 6). As the battery powering the sonde become depleted, the necessary 5 volts were no longer supplied to the sensor and caused the return voltage to be low. The low voltage is interpreted erroneously by the sonde as a higher temperature than actual (larger difference between reference and return voltages). This anomaly was expected and indicates that sonde configuration for the drifter application can take approximately 3100 samples using 6 alkaline C-cell batteries as a power supply.

The amperage draw by this configuration of the sonde spiked to 150 mA for one second while taking samples, resulting in a total consumption of approximately 7750 mAh over the entire deployment. The alkaline C-cell batteries used were capable of supplying approximately 46800 mAh [25], indicating that the majority of power consumed was during quiescence. Because quiescent power consumption of the drifter configuration is so high, this design is more suited for deployments less than 48 h .This could be extended by exploring alternative power supply options. For example the combination of rechargeable batteries and the addition of a solar panel would likely allow for longer deployments. However, the project that used this drifter design required deployments of no longer than 24 h, so emphasis was placed on minimizing cost. The moored application, as described here, attempted to extend the possible length of deployments. This was achieved by adding a power supply with higher ampere hour rating, eliminating unnecessary actions by the sonde, and using a quiescent state while not sampling. Similar to the power supply on the drifter, the resulting deployment duration was sufficient to meet the application requirements, so no further enhancements were made. Should future users want to extend the deployment duration further, additional design modifications will likely be necessary.

### 4.2. Drifter Deployment

Six drifters were released across the mouth of Mobile Bay in order to refine the logistics associated with the deployment and recovery of multiple units from a small (6.4 m) vessel, as well as test the performance of the frame and the overall functionality of the drifter as a whole. The drifter configuration design was found to be robust and reliable. The GPS data were able to transmit through the rubber cap consistently on all six drifters. Plots of the GPS data show that the drifters followed the flow of the ebbing, tidal current as expected. The collapsible design of the drifter frames allowed all six to be easily stowed on a 6.4 m long, center console boat. All the drifters were recovered eventually, however, drifter 10 was not located until the following morning, resulting in a deployment length of almost 24 h. The drifter 10 trajectory can be seen tracking a tidal ellipse over the course of its 24 h deployment (Figure 2). This is a strong indicator that wind forcing was minimized by the drifter frame design as intended. This frame design was found to be noticeably more efficient for deployment and transportation than traditional lagrangian drifter frames, while still satisfying the industry standard of 40:1 Drag Area Ratio [19].

It should be noted that, while temperature and salinity measurements are reported for this deployment in Figure 7, only minimal data validation was conducted as the purpose of this release was the refinement of the deployment and recovery logistics. However, a single temperature and salinity measurement was taken with a YSI 2030pro at the time of recovery for drifters 11–15, and the results are reported in Table 2. A comparison between the final drifter readings and the YSI 2030 indicate that drifters 13 and 14 reported salinity measurements outside of acceptable tolerance, while drifter 15 reported erroneous temperature measurements. Drifter 15’s issue was traced back to incorrect calibration offset entered in the programming code and not associated with instrument malfunction. Over the course of the deployments, the drifters exited the system on the ebb tide causing increases in the salinity as saltier Gulf of Mexico water is entrained into the outflow. Similarly, the temperatures increase with distance from the mouth, consistent with offshore conditions being slower in response to the seasonal cooling due to the larger water column.

### 4.3. Mooring Deployment

The modifications to the sonde design that were made to extend the deployment time, in order to accommodate the needs of the moored application, were sufficient. The two sondes that were deployed as part of the moored application design recorded temperature and salinity data for 24 consecutive days, and did not experience any “hanging” that prevented data collection or required a hard restart. Changes to the housing to allow for underwater deployment to depths up to 4 m resulted in no identifiable issues to the sonde or its power supply. Based the results presented here, the moored application successfully fulfilled the needs of the application, and is a legitimate low cost option for aquatic and/or marine physical monitoring efforts.

Results from the extended deployment period produced expected trends were consistent with expectations. The temperature and salinity measurements during this test deployment fluctuated diurnally with tidal cycles. The tidal influence on salinity is noticeably less defined at CT-2 when compared to CT-1 as a result of the geographic location of each site. CT-1, located at the mouth of a tributary leading into Mississippi Sound, experienced large fluctuations with each tidal cycle because of its proximity to the mouth of Mobile Bay where large salinity gradients are expected. In contrast, CT-2, located farther from sources of large salinity gradients, experienced lower overall salinities with less dramatic changes as a result of tidal advectance.

## 5. Conclusions

The primary objective of this project was to design a durable, reliable instrument capable of high resolution monitoring of physical properties in coastal marine systems, in response to the needs of two independent coastal applications. Additional emphasis was placed on minimizing cost, while maximizing customizability. The Arduino platform proved to be an exceptional tool for environmental monitoring, because of its surprising durability, low cost, and most importantly, the outstanding network of free online support. This can make high resolution monitoring projects a viable option for many educational and citizen science efforts. High school and undergraduate courses involving the construction, or deployment, of these instruments would provide valuable hands on experience that, until recently, was cost prohibitive.

While not as reliable and precise as top of the line instruments with designated user interfaces, software packages and technical support, the designs presented here do have a valid place in observational and functional ocean science as well as resource management. Researchers and graduate students are often forced to downsize projects as funding diminishes or becomes unavailable. Utilization of these low cost alternatives provides an avenue for new and innovative research by creating opportunities, where historically, few have existed. Furthermore, in coastal environments, where large changes in salinity can effect commercial important marine resources (e.g., Gulf of Mexico), these sonde designs provide a low cost option for state and local governments to develop or expand management capacity.

## Figures and Tables

**Table 1:** Individual and total component costs for the drifter and moored sonde designs.

**Drifter Application**			
Component	Manufacturer	Supplier	Cost ($)
Arduino Mega 2560	Arduino	Arduino.cc	45.95
Ultimate GPS Logger Shield	Adafruit	Adafruit.com	49.95
EZO-EC Microchip	Atlas Scientific	Atlas-scientific.com	58.00
Conductivity K 1.0 Probe	Atlas Scientific	Atlas-scientific.com	126.00
ENV-TMP Probe	Atlas Scientific	Atlas-scientific.com	25.00
PCB mount 3 pin 5.08 mm screw terminal	Uxcell	Amazon.com	1.90
		TOTAL COST	306.80
**Mooed Application**			
Component	Manufacturer	Supplier	Cost ($)
Arduino UNO	Arduino	Arduino.cc	24.95
Adafruit Data Logging Shield	Adafruit	Adafruit.com	19.95
EZO-EC Microchip	Atlas Scientific	Atlas-scientific.com	58.00
Conductivity K 1.0 Probe	Atlas Scientific	Atlas-scientific.com	126.00
ENV-TMP Probe	Atlas Scientific	Atlas-scientific.com	25.00
PCB mount 3 pin 5.08 mm screw terminal	Uxcell	Amazon.com	1.90
		TOTAL COST	255.80

**Table 2:** Deployment times, distances and final salinity measurements for six drifters released on 4 September 2014 across the mouth of Mobile Bay, Alabama.

Drifter	Release Time (GMT)	Recovery Time (GMT)	Total Deployment Time	Distance Traveled (km)	Drifter Final Salinity (ppt)	Drifter Final Temperature (°C)	YSI 2030 Salinity (ppt)	YSI 2030 Temperature (°C)
10	11:37	12:03	24 h 26 min	21.43	26.66	29.03	NA	NA
11	11:35	16:40	5 h 5 min	6.46	26.41	29.67	23.33	30.35
12	11:31	16:18	4 h 42 min	8.43	24.49	29.46	23.25	29.8
13	11:41	16.03	4 h 22 min	10.63	34.03	29.43	25.36	29.87
14	11:19	21:50	10 h 31 min	15.62	33.31	29.96	24.47	31.2
15	11:16	21:35	10 h 19 min	12.09	24.19	40.21	21.2	28.65

## Supplementary Materials

The source code for the drifter is available online at: https://github.com/glockridge/DrifterApplication. The source code for the moored application are available online at https://github.com/glockridge/MooredApplication.

## Abbreviations

The following abbreviations are used in this manuscript:

AUGPSS	Adafruit Ultimate GPS Logger Shield
Cm	Centimeter
CSV	Comma Separated Values
CT	Conductivity and temperature
DAR	Drag area ratio
DISL	Dauphin Island Sea Lab
DOAJ	Directory of open access journals
EC	Atlas Scientific EZO-EC electrical conductivity microchip
GMT	Greenwich Mean Time
GPS	Global Positioning System
GRIIDC	Gulf of Mexico Research Initiative Information & Data Cooperative
ICM	Integrated circuit microcontroller
ID	Inner diameter
IDE	Integrated development environment
LD	linear dichroism
Ma	Milliampere
MAh	Milliampere hours
MDPI	Multidisciplinary digital publishing Institute
Mega	Arduino Mega 2560
Mm	Millimeter
NMEA	National Marine Electronics Association
OD	Outer diameter
Ppt	Parts per thousand
PVC	Polyvinal chloride
RMS	Root Mean Square
RTC	Real time clock
SD	Standard deviation
SD	Secure digital memory card
SE	Standard error
TLA	Three letter acronym
Uno	Arduino Uno
WDT	Watchdog Timer
